# A comparison of a novel endoscopic “Su-Wang technique” with the open “Jaboulay’s procedure” for the surgical treatment of adult primary vaginal hydrocele

**DOI:** 10.1038/s41598-019-45229-5

**Published:** 2019-06-24

**Authors:** Junhao Lei, Chunhua Luo, Yangyang Zhang, Yuming Guo, Xinjun Su, Xinghuan Wang

**Affiliations:** 1 Department of Urology, Zhongnan Hospital of Wuhan University, Wuhan University, Wuhan, 430071 China; 2 Operating room, Department of Urology, Zhongnan Hospital of Wuhan University, Wuhan University, Wuhan, 430071 China; 3 Center for Evidence-based and Translational Medicine, Wuhan University, Wuhan, 430071 China

**Keywords:** Urogenital diseases, Urogenital diseases

## Abstract

This paper was aimed to introduce and compare outcomes of a novel “Su-Wang (S-W) technique” for endoscopic treatment of adult hydrocele with conventional open hydrocelectomy with “Jaboulay’s (JA) procedure” regarding adverse events (AEs) and patient satisfaction. In the randomized controlled trial, adult males with primary hydroceles were prospectively assigned into S-W or JA group. We recorded perioperative data and postoperative AEs (incision length, recurrence, hematoma, wound infection and edema vanished time). Finally, a total of 42 adult patients underwent the S-W (n = 22) or JA (n = 20) procedure. Procedures were successfully completed for all 42 patients. No significant differences were found between the two groups regarding age, symptom duration, body mass index, and size of the hydrocele. The incision length was significantly shorter in the S-W group (1.00 ± 0.24 cm) than in the JA group (6.10 ± 1.46 cm). After 6 months’ follow-up, complete data of 90.5% (38/42) were obtained. Severe AEs did not occur in any patient. Recurrence, hematoma, wound infection, edema vanished time values, and satisfaction in the S-W group were superior to those in the JA group. All patients in the S-W group were satisfied with this novel procedure, particularly due to the minimally invasive incision. In conclusion, the novel “S-W technique” for hydrocelectomy provided satisfactory cosmetic results with a 1-cm scrotal incision only. With the near-complete excision of the parietal TV, it resulted in no recurrence, fewer AEs, and rapid postoperative rehabilitation in comparison to the traditional “JA procedure.” The endoscopic “S-W technique” may be a viable alternative for the surgical treatment of adult primary vaginal hydrocele.

## Introduction

A hydrocele is an accumulation of fluid in the potential space between the visceral and parietal tunica vaginalis (TV) and is a common cause of scrotal swelling in adult males. Despite hydrocele being a benign condition, patients often seek medical intervention due to discomfort, cosmetic appearance, and limitations with daily activities. Various minimally invasive procedures have been widely applied for treatment, such as sclerotherapy^1^, endoscopic hydrocele ablation^2^, silicone catheter drainage^3^, and surgical methods, including the Jaboulay’s procedure^4^ or Lord’s technique^5^.

However, contemporary large series^6,7^ suggest that the overall complication rate of these procedures is as high as 20%. Complications involve infection, persistent swelling, hematoma, and pain. Some patients may also experience epididymal and/or vas deferens injuries, which may lead to decreased fertility^8^.

In order to minimize complications while maintaining comparable treatment success rates, we applied the novel “Su-Wang technique (S-W)” for endoscopic hydrocelectomy through a 1-cm scrotal skin incision, with near-complete excision of the parietal TV. We also compared the safety and clinical efficacy of the novel technique with those of the traditional “Jaboulay’s (JA) procedure” for adult primary vaginal hydrocele.

## Material and Methods

### Patients

Patients diagnosed with primary vaginal hydrocele were continuously recruited between September 2017 and August 2018 at Zhongnan Hospital of Wuhan University. The detailed medical history and complete physical examination of each patient were assessed before the inclusion of patients in the study. The diagnosis was confirmed by fluctuation and transillumination. Scrotal B-mode ultrasonography was also performed to exclude any other intrascrotal pathological conditions such as tumors, varicocele, inguinal hernia, etc.

The randomized controlled trial (RCT) was performed in accordance with the ethical principles of the Declaration of Helsinki. All patients provided written informed consent prior to inclusion. The study was approved by the Ethics Committee of Zhongnan Hospital of Wuhan University (Approval Number: 2017110). This was a registered clinical trial (Registration ID: ChiCTR-INR-17012652;Time: Sep. 12, 2017).

### Randomization

Randomization was performed prior to study commencement as follows. Opaque envelopes were sequentially numbered from 1 to 50. A table of random numbers was used for group assignment. If the last digit of the random number was an odd number, the patient was assigned to the Su-Wang group, and if the last digit was an even number, the patient was assigned to the Jaboulay group. The assignments were then placed into the opaque envelopes, and the envelopes were sealed. As eligible participants entered the trial, the envelopes were opened in a sequential order and each patient was assigned to a random group. The envelopes were opened by the operating surgeon after patient consent preoperatively.

### Surgical instruments and techniques

#### Su-Wang Group

**Figure 1:**
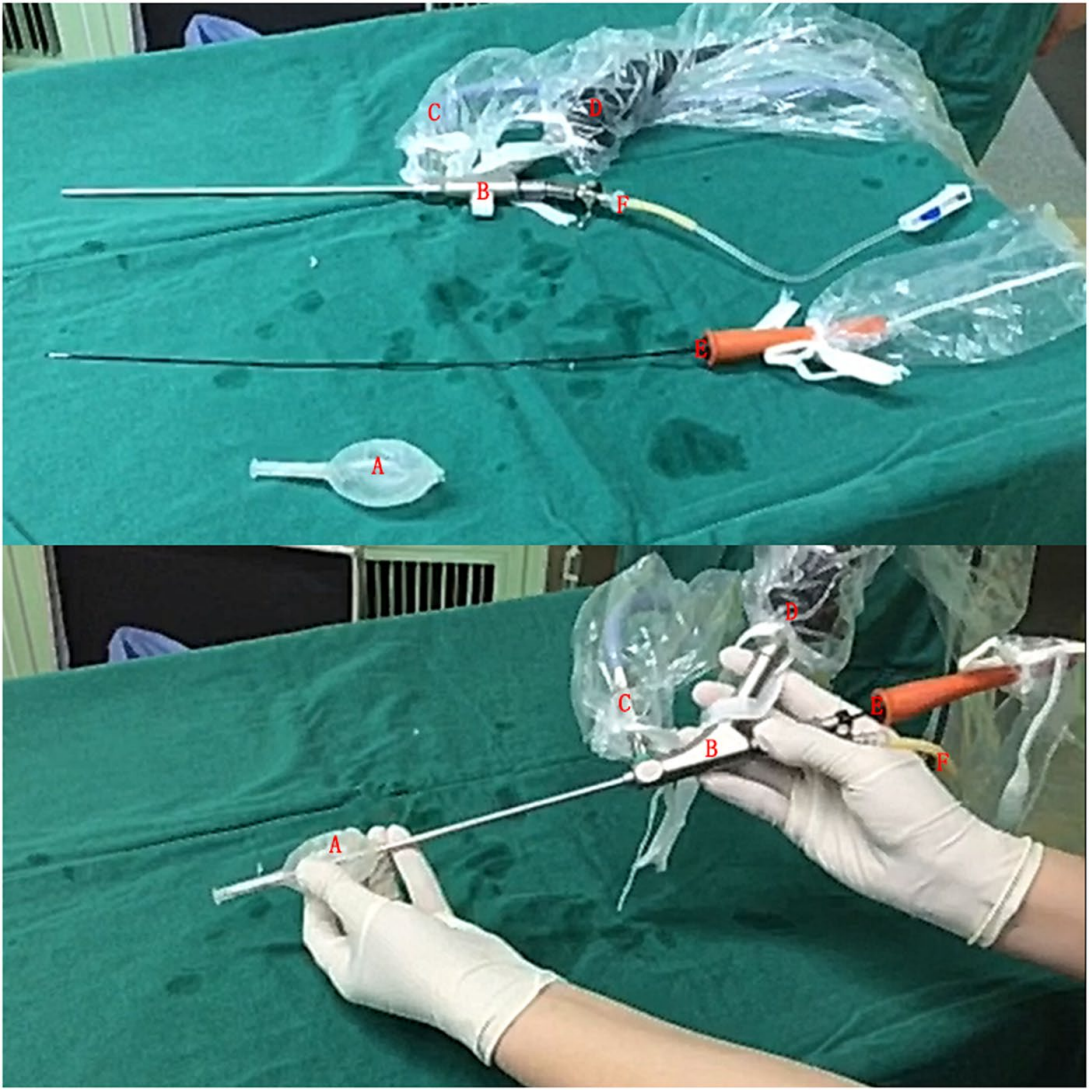
The instruments used for the “Su-Wang technique”. (**A**) The self-made working sheath. Its tail can insert into the vaginal cavity through the incision made at the scrotum, and can be steadily fixed to the parietal layer of the tunica vaginalis. Please also refer to Fig. 2b. Its cavity can be inserted by B. (**B**) AUTOKLAV (KARL STORZ, Germany) was used for scrotoscopy because no specialized devices were available. The device comprises a percutaneous mini-nephrolithotomy instrument set designed for the endoscopic treatment of kidney stones. It contains an endoscope and a working irrigation channel. (**C**) Connected to light source. (**D**) Connected to display screen. (**E**) Plasma cylindrical electrode (PCE). (**F**) Connected to the irrigation solution (normal saline).

Because no specialized devices were available, a mini-nephroscope (AUTOKLAV, KARL STORZ, Germany) was used for scrotoscopy^9,10^. The device comprises a percutaneous mini-nephrolithotomy instrument set designed for the endoscopic treatment of kidney stones. It contains an endoscope and a working irrigation channel (Fig. 1B). The instruments used for the “Su-Wang technique” are shown in Fig. 1.

**Figure 2:**
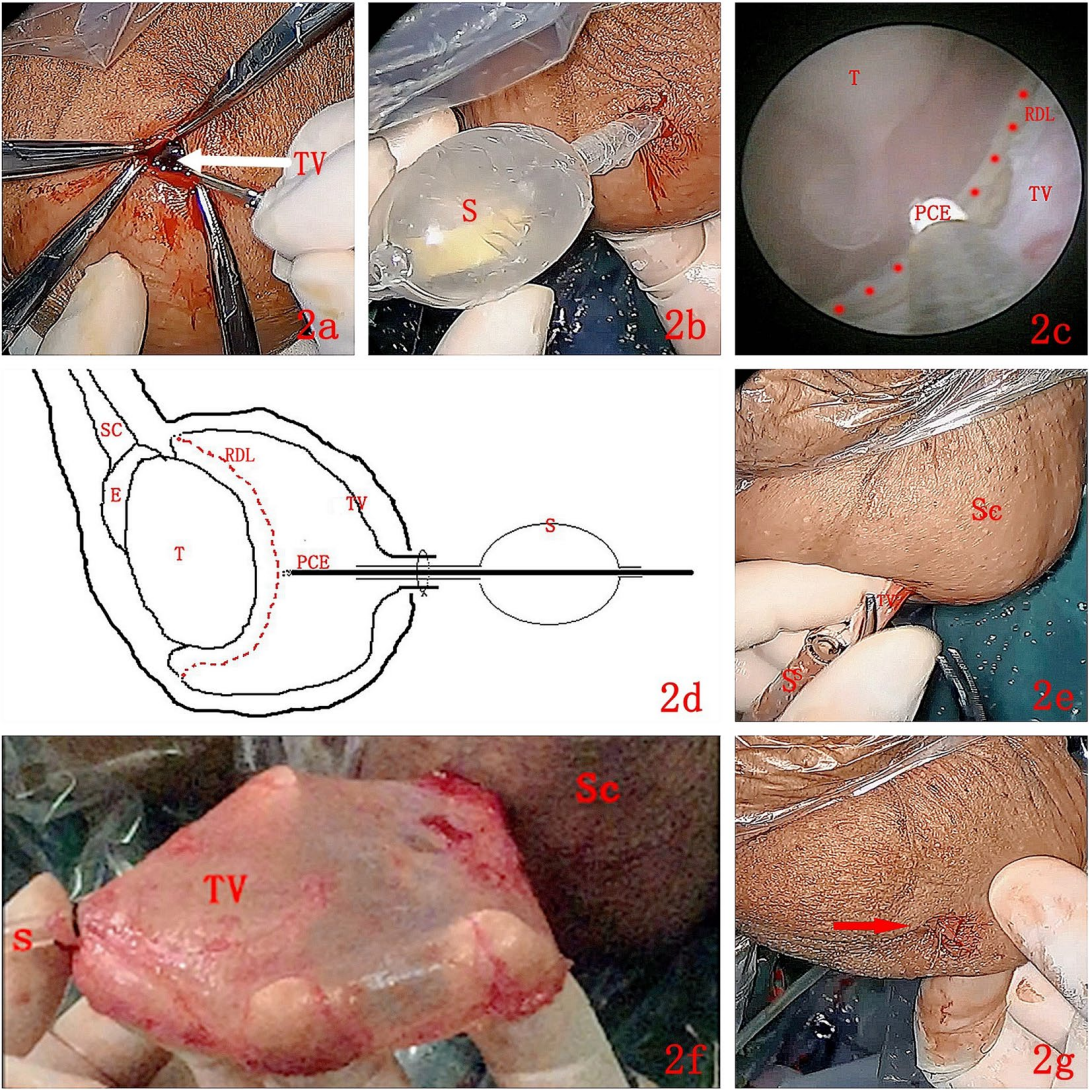
The main procedures for the “Su-Wang technique”. (**a**) TV: Tunica vaginalis was shown with a white arrow. A transverse incision of approximately 1 cm in length was made at the anterior, superior part of the scrotum. Then, the skin, dartos, and superficial fascia of the scrotum were cut layer by layer, until the parietal TV was exposed. (**b**) S: Sheath. The tail of the self-made working sheath was passed through the incision into the cavity of the TV, steadily fixed at the parietal layer. (**c**,**d**) The endoscopic view and schematic diagram of “Su-Wang technique”. T: Testis. TV: Tunica vaginalis. PCE: Plasma cylindrical electrode. RDL: The Red Dotted Line indicated the marker line. SC: Spermatic cord. E: Epididymis. S: Sheath. A PCE was used to burn a circle at the parietal TV to serve as a marker. The distance between the burned area and the testis or epididymis was 1–2 cm. The parietal TV was burned along the circular marker with the PCE. The burning depth penetrated the parietal TV only, without reaching the deep fascial layer of the scrotal wall. (**e**,**f**) Sc: Scrotum. TV: Tunica vaginalis. S: Sheath. The adhesion between the parietal TV and the deep fascial layer of the scrotal wall was bluntly separated, stripping the parietal TV completely. During the dissection procedure, electrocoagulation was used to stop bleeding when necessary. (**g**) An incision about 1 cm long was left in the scrotum immediately after the procedures (shown with a red arrow).

First, a transverse incision of approximately 1 cm in length was made at the anterior, superior part of the scrotum. Then, the skin, dartos, and superficial fascia of the scrotum were cut layer by layer, until the parietal TV was exposed. An incision of approximately 0.5 cm in length was made, and then a self-made working sheath was passed through the incision into the cavity of the TV, steadily fixed at the parietal layer. Through the self-made working sheath, the scrotoscope was inserted into the vaginal cavity. Continuous irrigation (0.9% NaCl) was used to distend the vaginal cavity and provide a clear endoscopic vision. A plasma cylindrical electrode was used to burn a circle at the parietal TV to serve as a marker. The distance between the burned area and the testis or epididymis was 1–2 cm. The parietal TV was burned along the circular marker with the plasma cylindrical electrode. The burning depth penetrated the parietal TV only, without reaching the deep fascial layer of the scrotal wall. After the burning procedure, the scrotoscope was pulled out. Subsequently, the adhesion between the parietal TV and the deep fascial layer of the scrotal wall was bluntly separated, stripping the parietal TV completely. During the dissection procedure, electrocoagulation was used to stop bleeding when necessary. Finally, a drain was left in place. The key steps of the Su-Wang techniques are shown in Fig. 2.

#### Jaboulay Group

The traditional Jaboulay’s procedure was performed according to the previously published article by Jaboulay^4^. All procedures were performed in the inpatient setting, and patients were discharged only when the wound was healed. To decrease bias, all procedures were performed by a single skilled surgeon (SXJ).

### Observational indexes and endpoints

The size of the hydrocele, operation time, incision length, intraoperative blood loss, hospital stay, and adverse events (AEs) were recorded in both groups. The size of the hydrocele was recorded immediately after the parietal TV was exposed by aspirating the fluid with a 50-mL syringe. The operation time was defined as the duration from the first incision to the last suture. Intraoperative blood loss in the JA group was calculated based on the weight increase of the standard gauze (1 g was considered as approximately 1 ml of blood loss). Recurrence was considered as the primary endpoint, defined as a clinically detectable characteristic swelling in the scrotum confirmed by B-mode ultrasound at 6 months postoperatively. The secondary endpoints were postoperative hematoma, wound infection, and edema vanished time. Edema vanished was defined as the volume of the affected side having recovered to the status of the unaffected side. Based on the Clavien/Dindo classification, The severity of each AE was graded into five levels: Grade I—Grade V^11^. Patients’ satisfaction with the procedure and AEs were collected by another urologist who was blinded to the type of treatment. Satisfaction assessment was based on their subjective feeling to the following two aspects: cosmetic effect of the scrotum appearance and whether the operation brought about any trouble to the patients (i.g. wound infection, hemorrhage). Last follow-up visits were conducted at 6 months postoperatively at the outpatient clinic.

### Statistical analysis

All statistical analyses were performed using a statistical software package (SPSS, Version 16.0, Chicago, IL, USA). Continuous variables were expressed as mean ± standard deviation. Discrete variables were expressed as percentages. The *t*-test was used to compare continuous data, and chi-square tests were used for discrete data. Statistical significance was defined as p < 0.05 (two-sided). Two authors (LJH and LCH) independently conducted the statistical analysis, and all disagreements were resolved through discussion among the study group members.

## Results

A total of 42 adult patients with an average age of 60.05 ± 13.67 years (range: 33–85) underwent the S-W (n = 22) or JA (n = 20) procedure. Procedures were successfully completed for all 42 patients. Although some researchers^12^ have performed hydrocelectomy in an outpatient setting, we recommended 3 days of hospitalization in case of any unexpected AEs.

**Figure 3:**
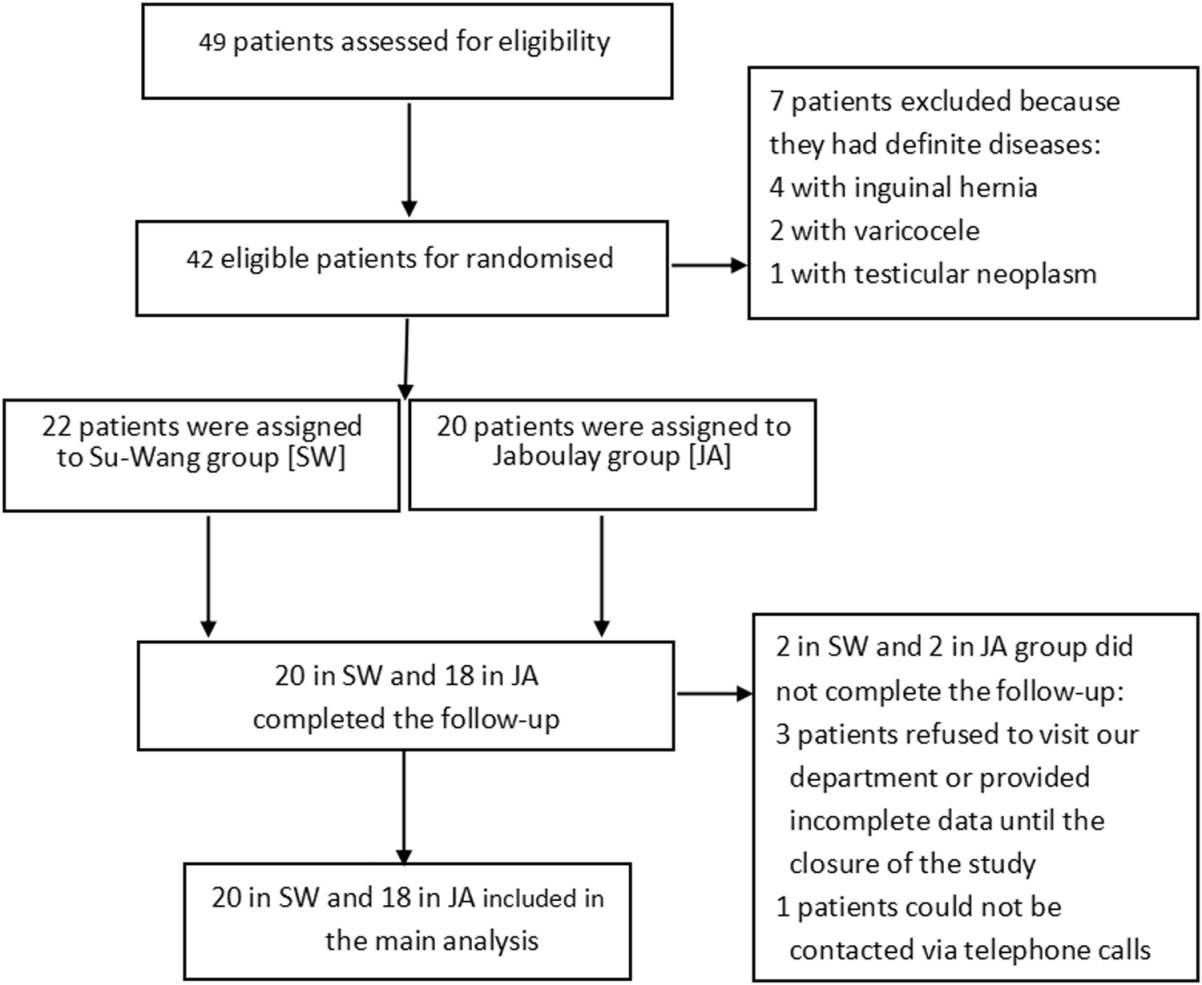
The CONSORT flowchart of the patients through each stage of the study.

After 6 months of follow-up, the complete follow-up data of 90.5% (38/42) of the patients (20 in the S-W group and 18 in the JA group) were obtained at the outpatient clinic. The CONSORT flowchart of the patients throughout each stage of the study is shown in Fig. 3.

No significant differences were found between the two groups regarding age, symptom duration, body mass index, and size of the hydrocele. The operation time of the S-W and JA groups was 55.50 ± 6.26 min and 46.82 ± 7.95 min, respectively (p < 0.001). The incision length was significantly shorter in the S-W group (1.00 ± 0.24 cm) than in the JA group (6.10 ± 1.46 cm). Recurrence, hematoma, wound infection, and edema vanished time values in the S-W group were superior to those in the JA group. All patients in the S-W group were satisfied with this novel procedure, particularly due to the minimally invasive incision. Detailed baseline, preoperative, and follow-up data are shown in Table 1.

**Table 1:** Baseline, perioperative and follow-up data when compared “Su-Wang technique” with “Jaboulay’s procedure” for adult primary vaginal hydrocele.

Items	SW (n = 22)	JA (n = 20)	P (2-sided)
**Baseline data**			
Age (years)	60.00 ± 13.86	60.10 ± 13.80	0.981
Body mass index (BMI)	24.03 ± 3.98	24.14 ± 2.88	0.917
Symptom duration (mons)	12.27 ± 25.00	13.00 ± 23.82	0.924
Size of hydrocele (ml)	142.98 ± 100.15	136.57 ± 88.06	0.827
**Perioperative data**			
Blood loss (ml)	NA	12.15 ± 5.11	NA
Operation time (min)	55.50 ± 6.26	46.82 ± 7.95	<0.001
Incision length (cm)	1.00 ± 0.24	6.10 ± 1.46	<0.001
Hospital stay (days)	3	3	—
Return to daily activities (days)	3.00 ± 1.08	5.72 ± 1.53	<0.001
**Follow-up data**			
Recurrence	0/20*	4/18*	0.041^&^
Wound infection	0/20	1/18	0.474^&^
Hematoma	0/20	2/18	0.218^&^
Edema	6/20	13/18	0.022
Edema vanished time (days)	2.50 ± 1.19	4.56 ± 1.46	<0.001
Satisfaction percent (%)	100% (20/20)	83.3% (14/18)	0.041^&^

SW: Su-Wang technique. JA: Jaboulay’s procedure. Data are showed as means ±  standard deviations. P< 0.05 was considered to reflect a significant difference. NA: Not applicable. *Two cases in the SW group and two cases in the JA group could not be connected during follow-up, so the total patients that providing the follow-up data were 20 and 18 cases for each group. & Fish’s Exact Test was used for the comparisons in this table because total N = 38 < 40.

Severe AEs did not occur in any patient. In the JA group, 4 patients occurred recurrence and reoperations were applied to 3 of them; another one refused to go back for intervention. One patient had a wound infection and recovered after antibiotic application for 1 week and wound dressing. No wound infection occurred in the S-W group. 6 patients in the S-W group and 13 patients in JA group experienced edema. No hematoma occurred in the S-W group, but 2 patients experienced hematoma in the JA group. All edemas and hematomas recovered spontaneously without any intervention in less than 1 week. Based on the Clavien/Dindo classification, the severity of each AE and their outcomes are shown in Table 2.

**Table 2:** The severity grading ^&^ of adverse events occurred in “Su-Wang” and “Jaboulay” groups.

AEs^$^	SW			JA		
	Grade I	Grade II	Grade III	Grade I	Grade II	Grade III
Wound infection (N = 1)	—	—	—	—	1^#^	—
Edema (N = 19)^	6	—	—	13	—	—
Hematoma (N = 2)^	0	—	—	2	—	—

^&^The severity of each AE was graded based on the Clavien/Dindo classification. ^#^One case with wound infection recovered after the application of antibiotics for one week and wound dressing. ^^^All the cases suffered edema or hematoma recovered spontaneously without any intervention less than one week. ^$^Adverse events. Grade I: Any deviation from the normal postoperative course without the need for pharmacological treatment or surgical, endoscopic, and radiological interventions. Allowed therapeutic regimens are: drugs as antiemetics, antipyretics, analgetics, diuretics, electrolytes, and physiotherapy. This grade also includes wound infections opened at the bedside. Grade II: Requiring pharmacological treatment with drugs other than such allowed for grade I complications. Blood transfusions and total parenteral nutrition are also included. Grade III: Requiring surgical, endoscopic or radiological intervention. Grade IIIa Intervention not under general anesthesia; Grade IIIb Intervention under general anesthesia. Grade IV: Life-threatening complication requiring IC/ICU management. Grade IVa Single organ dysfunction (including dialysis); Grade IVb Multiorgan dysfunction. Grade V: Death of a patient.

## Discussion

Regarding the etiology of adult hydrocele, an imbalance between the secretory and absorptive capacities of the TV is responsible for fluid collection between the parietal and visceral layers of the TV. Current treatments aim to decrease or eliminate this secretory function of the TV. Traditional percutaneous aspiration usually leads to a high recurrence rate; thus, the use of more than one instillation of sclerosing agents may be needed in many cases^13,14^. Although hydrocelectomy is considered as the gold standard for the surgical resolution of hydrocele, it usually leads to a high rate of scrotal hardening^15,16^. Therefore, the development of new procedures with fewer complications, high success rate, and patient satisfaction is becoming urgent.

Recently, several minimally invasive techniques have been developed for treating hydroceles, particularly for the attempt of endoscopic hydrocelectomy. To the best of our knowledge, the first described endoscopic hydrocelectomy was reported by Ho *et al*. in 1992^17^, and despite being a single case only, it should be considered groundbreaking in the surgical treatment of adult hydrocele. In the present study, we describe the novel endoscopic “S-W technique” for minimal access hydrocelectomy performed through a 1-cm scrotal incision for the treatment of a large hydrocele (main size of approximately 143 ml). Based on our data, the innovative “S-W technique” resulted in no recurrence, fewer complications, and higher patient satisfaction in comparison to the traditional “JA procedure.”

The most exciting result of the S-W group was the near-perfect level of satisfaction in comparison to the JA group (100% vs 83.3%). In the latter group, some patients complained that the incision was too long (6.10 ± 1.46 cm), resulting in poor cosmetic results. Although the S-W technique had a longer operation time (S-W vs. JA: 55.50 ± 6.26 vs 46.82 ± 7.95 min, p < 0.001), it required a 1-cm scrotal skin incision only. In the follow-up period, the small incision was difficult to detect if not closely observed. Furthermore, due to minimal invasion and fewer AEs, patients in the S-W group spent a shorter time recovering before returning to daily activities in comparison to those in the JA group (3.00 ± 1.08 vs 5.72 ± 1.53 days).

Regarding the primary endpoint, there was no recurrence in S-W group (0/20) during the 6 months of follow-up, which was statistically lower than that of the JA group(4/18). In another endoscopic hydrocelectomy study by Emir *et al*.^18^, the authors reported a recurrence rate of 7.4% (2/27). They used a 27 F resectoscope with a 30-degree lens for scrotoscopy and a cylindrical electrode. The greatest difference between the method of Emir *et al*. and the S-W method was the extent of surgical intervention around the parietal TV. Emir *et al*. coagulated all surfaces of the parietal TV, whereas we removed almost the entire parietal TV. Whether the parietal TV should be fully cauterized needs further discussion. A TV that is not fully cauterized would still secrete fluid continuously into the sac and may be responsible for recurrence. Although there was no recurrence in the S-W group, we need to be careful with drawing conclusions before the results of the long-term follow-up are known.

The most common complication in the S-W group was scrotal edema [30% (6/20)]. Nonetheless, the incidence of edema was significantly lower than that in the JA group (72%, 13/18). However, the edema vanished time in the S-W group was significantly shorter than that in the JA group (2.50 ± 1.19 vs 4.56 ± 1.46 days). Longer vanished times in the JA group might have been attributed to excessive handling and wide dissection of the hydrocele sac, particularly in cases of hydrocele sacs of larger sizes (142.98 ± 100.15 vs 136.57 ± 88.06 ml). Edema in the S-W group might also be related to the leakage of irrigation solution around the scrotal wall occurring when the burning depth penetrated beyond the parietal TV. With the improvement of the surgeon’s skills, we found that regarding the severity and incidence of edema, the outcomes of patients recruited at a later stage were superior to those of patients recruited in the initial stage.

Postoperative hematoma did not occur in any patient in the S-W group. The novel “S-W technique” omitted the dissection of the parietal TV from the deep fascial layer of the scrotal wall under direct vision, facilitating easier use of electrocoagulation to stop bleeding if necessary during the dissection procedure. In addition, the burning process around the circular marker was in fact a process of preliminary hemostasis, a key difference in comparison to the “Pull-Through Technique” published by Önol *et al*. in 2009^19^. Using the latter method, it would be very difficult to stop bleeding, specifically around the edge of the residual TV because the edge can easily retract to the scrotum when the hydrocele sac is excised by an electrocautery at its base. Another advantage of the “S-W technique” was that it decreased the stretching force applied to the testis and the epididymis when the hydrocele sac was excised by an electrocautery at its base because the hydrocele sac had been disconnected from the testis and the epididymis. These might be the reasons why there was no occurrence of hematoma in the S-W group. In contrast, a certain percentage of hematomas, although not evident in any case, was reported by Önol *et al*.

Finally, our study had several limitations. Although our data were in favor of the S-W technique, 6 months of follow-up might be too short to reveal potential complications and recurrence might occur during long-term follow-up. Patients operated with the “Pull-Through Technique” in the study of Onol *et al*.^19^ were followed-up for a minimum of 18 months, and one patient was admitted to the hospital with a recurrence of the hydrocele after 30 months of follow-up. Moreover, the small sample size of this study remained as a limitation to reveal the difference of AEs between the two methods. Randomized controlled trials with large-scale, multi-center, long-term, and rigorous design would provide more robust conclusions.

## Conclusions

The novel “S-W technique” for hydrocelectomy provided satisfactory cosmetic results with a 1-cm scrotal incision only. With the near-complete excision of the parietal TV, it resulted in no recurrence, fewer AEs, and rapid postoperative rehabilitation in comparison to the traditional “JA procedure.” The endoscopic “S-W technique” may be a viable alternative for the surgical treatment of adult primary vaginal hydrocele^20^.
